# The health and cost implications of high body mass index in Australian defence force personnel

**DOI:** 10.1186/1471-2458-12-451

**Published:** 2012-06-19

**Authors:** Jonathan Peake, Susan Gargett, Michael Waller, Ruth McLaughlin, Tegan Cosgrove, Gary Wittert, Peter Nasveld, Peter Warfe

**Affiliations:** 1 The University of Queensland, Centre for Military and Veterans’ Health, Brisbane, Australia; 2 The University of Queensland, School of Human Movement Studies, Brisbane, Australia; 3 The University of Queensland, Centre of National Research on Disability and Rehabilitation Medicine, Brisbane, Australia; 4 School of Medicine, The University of Adelaide, Adelaide, Australia; 5 The University of Queensland, Centre for Military and Veterans’ Health, Herston QLD 4006, Australia

## Abstract

**Background:**

Frequent illness and injury among workers with high body mass index (BMI) can raise the costs of employee healthcare and reduce workforce maintenance and productivity. These issues are particularly important in vocational settings such as the military, which require good physical health, regular attendance and teamwork to operate efficiently. The purpose of this study was to compare the incidence of injury and illness, absenteeism, productivity, healthcare usage and administrative outcomes among Australian Defence Force personnel with varying BMI.

**Methods:**

Personnel were grouped into cohorts according to the following ranges for (BMI): normal (18.5 − 24.9 kg/m^2^; n = 197), overweight (25–29.9 kg/m^2^; n = 154) and obese (≥30 kg/m^2^) with restricted body fat (≤28% for females, ≤24% for males) (n = 148) and with no restriction on body fat (n = 180). Medical records for each individual were audited retrospectively to record the incidence of injury and illness, absenteeism, productivity, healthcare usage (i.e., consultation with medical specialists, hospital stays, medical investigations, prescriptions) and administrative outcomes (e.g., discharge from service) over one year. These data were then grouped and compared between the cohorts.

**Results:**

The prevalence of injury and illness, cost of medical specialist consultations and cost of medical scans were all higher (p < 0.05) in both obese cohorts compared with the normal cohort. The estimated productivity losses from restricted work days were also higher (p < 0.05) in the obese cohort with no restriction on body fat compared with the normal cohort. Within the obese cohort, the prevalence of injury and illness, healthcare usage and productivity were not significantly greater in the obese cohort with no restriction on body fat compared with the cohort with restricted body fat. The number of restricted work days, the rate of re-classification of Medical Employment Classification and the rate of discharge from service were similar between all four cohorts.

**Conclusions:**

High BMI in the military increases healthcare usage, but does not disrupt workforce maintenance. The greater prevalence of injury and illness, greater healthcare usage and lower productivity in obese Australian Defence Force personnel is not related to higher levels of body fat.

## Background

In the civilian population, high body mass index (BMI) is strongly linked with increased prevalence of acute and chronic illness [1,2] and injury [3,4], healthcare costs, disability and absenteeism [5,6]. Many of these relationships are also evident within military populations [7-10]. Past research on the health, administrative and economic consequences of high BMI in military populations has been conducted in the United States [7,11-14], Europe [8,10,15] and Israel [16], and may partially apply to Australian Defence Force (ADF) personnel. However, compared with military populations in these geographic regions, the ADF employs different standards of body composition and physical fitness for recruitment, and faces different operational demands. The health, administrative and economic consequences of high BMI specifically within the ADF therefore require more detailed investigation. To obtain a complete and informative understanding of the health, administrative and economic consequences of high BMI in military populations, it is necessary to evaluate these factors together, rather than in isolation. Military service is unusual compared with many other professions, because it requires a greater level of physical health. High BMI in the military can potentially disrupt military readiness, workforce maintenance and productivity—all of which are integral to effective military service. Most research on the effects of high BMI in military populations has not delineated the effects of increased levels of body fat. This factor is important to consider because of the limitations of BMI as a measure of body composition [17-19].

Historically, the ADF has recruited applicants with a BMI of ≤30 kg/m^2^. In 2006, the ADF revised this standard to accept applicants with BMI ≥30 kg/m^2^ who meet all other health standards. This decision was made because limitations on standards of entry were restricting the number of potential applicants to the ADF. The purpose of this study was to evaluate this policy change by comparing the incidence of injury and illness, absenteeism, productivity, healthcare usage and administrative outcomes among Australian Defence Force personnel in normal, overweight and obese categories of BMI.

## Methods

### Data collection

This study was approved by the Australian Defence Health Research Ethics Committee and the Human Research Ethics Committee at The University of Queensland. This was a retrospective cohort study involving 679 ADF male (n = 597) and female (n = 82) personnel from army, navy and air force service branches. Reservists were not included in the study. Data were collected only during the first 12 months of training, or up until the date of discharge (whichever occurred earliest). Not all personnel joined the ADF at the same time, so the study was conducted over approximately 2 years between 2008–2010. A clinical and administrative audit was used to collect data from ADF Central Medical Records and Directorate of Workforce Information. Individuals were eligible if they passed the requirements for entry into ADF, and presented with a BMI ≥18.5. Eligible individuals were identified by recruitment officers, who then provided the personal details of each individual to the ADF. We then retrieved the personal medical records for these individuals.

After receiving the personal medical record for each individual, we classified each individual into one of four cohorts, based on their individual BMI. The first two cohorts included ‘normal’ individuals with BMI 18.5 − 24.9 kg/m^2^ (n = 197) and ‘overweight’ individuals with 25–29.9 kg/m^2^ (n = 154). The other two cohorts included ‘obese’ individuals with BMI ≥30 kg/m^2^ and either restricted body fat (≤28% for females, ≤24% for males) (n = 148) or no restriction on body fat (n = 180). Body fat was assessed during an Entry Level Medical Examination conducted by trained staff at ADF recruiting centres. Body fat was evaluated by measuring neck and abdominal circumference for males, and neck, abdominal, biceps, forearm and thigh circumference for females. The ADF uses these circumference measurements to assess body fat for reasons of convenience and ease of practice.

Data relating to injury, illness and healthcare usage (e.g., frequency and duration of hospital visits, consultations with medical practitioners, medical scanning procedures and pharmaceutical prescriptions) were obtained from ADF Central Medical Records. Injuries were broadly classified as new injuries, recurring injuries, or chronic (i.e., pre-existing) injuries. The cause of injuries was classified as a result of physical training, overuse, or stumbling/falling/tripping. Injuries were also classified as mild, moderate and severe injuries, or injuries requiring no treatment or more/less than five days of incapacity. Data relating to administrative outcomes (e.g. restricted work days, sick leave, reclassification of Medical Employment Classification, all-cause discharge) were obtained from the Directorate of Workforce Information within the ADF. Medical Employment Classification is a standard of health as assessed by medical personnel. It ranges on a scale from 1 (medically fit, without restriction, for deployment for a particular military occupation) to 4 (medically unfit for deployment for more than 12 months and requires review by a Medical Employment Classification Review Board).

### Economic modelling

To calculate the economic cost of lost productivity, estimated daily wage rates were multiplied by the number of days that individuals were unable to perform ‘normal duties’ as a result of injury or illness. The time lost from normal duties was classified into the number of days the ADF member was placed on restricted duties (restricted work days) and the number of days they were unable to perform any duties (full days off work). Productivity losses were then compared between the normal, overweight and obese cohorts. To calculate estimates of daily wage rates, base salaries for permanent ADF members as of November 2009 were used. The base salary rate for each ADF rank was used for the analysis, and included the Service Allowance. Wages per calendar day are a reasonable measure of the cost of productivity losses (to the ADF) if the time lost (i.e., the recorded data on the number of restricted work days, full days off work and hospital days) incorporates both work and non-work days (e.g., weekends, off-duty days). However, wages per calendar day will underestimate the cost if the data for time lost refers only to work time/days. Therefore, a sensitivity analysis was conducted using estimates of wages per work-day as the unit cost measure of productivity losses. The use of the two unit cost measures provides upper- and lower-bound estimates of the cost of lost productivity to the ADF. The ‘wages per calendar day’ figure may underestimate the real cost, whereas the ‘wages per work-day’ figure may overestimate the real cost. The real productivity cost is considered to lie somewhere between the two.

The estimated wages per calendar day for injured or sick ADF members were used to calculate the cost of full days off work and hospital days. For restricted work days, it was assumed that ADF members placed on restricted work days were able to perform 50% of their normal duties. Therefore, 50% of their wages per (calendar) day were used as the cost of restricted work days.

The unit cost data used in this study, to determine estimates of the various costs to the ADF, pertain to different financial years. To account for this, the unit cost data were converted into Australian 2009–10 dollars. Specifically, the Implicit Price Deflator for ‘General Government – National; Final Consumption Expenditure – Non-defence’ (Australian Bureau of Statistics 2010) [20] was used to convert 2007–08 and 2008–09 dollars into 2009–10 dollars. To account for inflation, 2007–08 dollars were multiplied by 1.05, and 2008–9 dollars were multiplied by 1.02.

The cost of hospital stays was estimated from the National Hospital Cost Collection *Cost Report Round 12 (2007–2008)* September 2009 [21]. The cost of hospital stays was estimated at $1,324 per day by dividing the average cost per public separation (including depreciation) of $3,907 by the average length of stay (2.95 days).

The costs of medical consultations, scans and pathology investigations were obtained from the Australian Government Department of Health and Ageing ‘Medical Benefits Schedule Online Medicare Benefits Schedule’ [22]. We consulted a general physician for more specific information and clarification of the cost of individual items on this schedule. Scans included X-ray, ultrasound, magnetic resonance imaging and computed tomography procedures. Pathology investigations included a wide range of clinical tests on biological samples (e.g., blood, urine, swabs) for liver, kidney and thyroid functions, infections, diseases, iron status etc. The Australian Government Department of Veterans Affairs adjusts the cost of items on the Medical Benefits Schedule for ADF personnel. We obtained these adjustment rates from the Australian Government Department of Veterans Affairs Fee Schedule for Medical Services [23]. Most individuals visited one or more medical practitioners on several occasions. The total cost of medical consultations was calculated for each individual, and this figure was used for data analysis. Most individuals also required more than one scan or pathology investigation. The total costs of scans or pathology investigations were calculated for each individual, and these figures were used for data analysis. Data for medical consultations were screened to identify initial and subsequent consultations, and different fees were used for initial versus subsequent consultations.

The costs of scans and pathology investigations were obtained from the Australian Government Department of Health and Ageing ‘MBS Online Medicare Benefits Schedule’ at [22]. Although ADF members were able to visit medical practitioners privately at their own discretion, the costs of such consultations were not recorded in their medical records, and have not been included in the current analysis.

### Data analysis

Data for counts of injuries, illness, restricted work days, sick days and hospital visits were compared between the cohorts using negative binomial regression. Numbers of participants, a change in Medical Employment Classification and discharge within the first year were compared using Chi-squared tests. Productivity, hospital and medical consultation costs were compared between groups using Kruskall-Wallis test for overall cohort differences, and Wilcoxon’s rank test for differences between two cohorts. Statistical analyses were performed using SAS version 9.2 (SAS Institute Inc, Cary NC) and Stata 10.0 (StataCorp. 2007. *Stata Statistical Software: Release 10*. College Station, TX). Statistical significance was set at α = 0.05.

## Results

### Demographics

At the time of recruitment, age, sex, service and Medical Employment Classification varied significantly between the four cohorts (Table 1). In the normal cohort, most individuals (65%) were aged ≤19 years. In contrast, the proportion of individuals in the overweight and obese cohorts was more evenly distributed across the age categories. Most individuals (95%) in all four cohorts entered the ADF with Medical Employment Classification 1 (medically fit, without restriction, for deployment for a particular military occupation).

**Table 1:** Cohort characteristics

	**Cohort**				
	**Normal**	**Overweight**	**Obese Restricted body fat**	**Obese No restriction on body fat**	** *p* -value**
	**n = 197**	**n = 154**	**n = 148**	**n = 180**	
	**n (%)**	**n (%)**	**n (%)**	**n (%)**	
Sex					
Male	160 (81)	136 (88)	140 (95)	161 (89)	0.003
Female	37 (19)	18 (12)	8 (5)	19 (11)	
Service					
Navy	63 (32)	51 (33)	40 (27)	74 (41)	0.002
Army	90 (46)	70 (45)	78 (53)	86 (48)	
Air Force	44 (22)	33 (21)	30 (20)	20 (11)	
Age group					
≤19	126 (65)	54 (35)	54 (36)	59 (33)	<0.0001
20–24	56 (29)	51 (33)	49 (33)	59 (33)	
25–29	2 (1)	28 (18)	20 (14)	31 (17)	
≥30	10 (5)	21 (14)	25 (17)	31 (17)	
Rank					
Officer	20 (13)	8 (7)	7 (5)	11 (7)	0.06
Recruit	139 (87)	115 (94)	131 (95)	150 (93)	
MEC status					
1	139 (95)	118 (91)	107 (85)	142 (88)	0.02
2	2 (1)	10 (8)	4 (3)	9 (6)	
201	0 (0)	2 (2)	1 (1)	4 (2)	
202	2 (1)	7 (5)	2 (2)	5 (3)	
203	0 (0)	1 (1)	1 (1)	0	
3	5 (3)	1 (1)	14 (11)	10 (6)	
4	0 (0)	0	1 (1)	0	

N.B. MEC = Medical Employment Classification.

MEC 1 = medically fit, without restriction, for deployment for a particular military occupation.

MEC 2 = medically fit for deployment but with limitations on the range of duties or geographic location, and/or a requirement for access to various levels of health logistic or personnel support.

MEC 3 = medically unfit for deployment in the medium term (up to 12 months).

MEC 4 = medically unfit for deployment for more than 12 months and requires review by a Medical Employment Classification Review Board.

MEC data were unavailable for a small number of participants in each cohort.

### Illness and injuries

Data for the prevalence of injury and illness were adjusted for age (15–19, 20–24, 25–29, 30+ years), sex, service (i.e., army, navy, air force) and rank (i.e., officer, recruit). To perform this adjustment, we added age, sex and service into the statistical models. Injuries were significantly more prevalent in the overweight cohort (40%) and both the obese cohorts (50 − 60%) compared with the normal cohort (Table 2). The prevalence of illness was also significantly greater in both the obese cohorts (30 − 40%) compared with the normal cohort (Table 2). The prevalence of all injuries combined and illness was similar between the obese cohorts.

**Table 2:** Prevalence of injury and illness

	**Cohort**			
	**Normal**	**Overweight**	**Obese Restricted body fat**	**Obese No restriction on body fat**
	**n = 197**	**n = 154**	**n = 148**	**n = 180**
Injuries per person	1.5	2.1	2.2	2.4
Ratio of means (95% CL)	1 (Reference)	1.4 (1.1, 1.8)	1.5 (1.2, 1.9)	1.6 (1.3, 2.1)
*p*-value	—	0.007	0.001	<0.001
Illnesses per person	3.7	4.1	4.7	5.0
Ratio of means (95% CL)	1 (Reference)	1.1 (0.9, 1.4)	1.3 (1.0, 1.6)	1.4 (1.1, 1.7)
*p*-value	—	0.24	0.02	0.002

N.B. Data are adjusted for age, sex, service and rank. CL, confidence limits.

New injuries (2.0 − 2.7×), mild injuries (2.7 − 3.0×) and injuries that required absence from work (2.9 − 3.5×) were significantly more frequent in both obese cohorts compared with the normal cohort (Table 3). Injuries resulting from physical training (3×), overuse injuries (4.1×), injuries resulting in incapacity for <5 days (6.7×), injuries requiring absence from physical training (1.8×) and injuries requiring absence from work (2.9×) were also significantly more frequent in the obese cohort with restricted body fat compared with the normal cohort (Table 3). Compared with the obese cohort with restricted body fat, the prevalence of injuries from physical training (rate ratio = 0.4; 95% CL 0.2, 0.8; p = 0.003), overuse injuries (rate ratio = 0.4; 95% CL 0.2, 0.9; p = 0.009) and injuries resulting in incapacity for <5 days (rate ratio = 0.4; 95% CL 0.1, 1.0; p = 0.03) was significantly lower in the obese cohort with no restriction on body fat (Table 3).

**Table 3:** Injury characteristics

	**Cohort**			
	**Normal**	**Overweight**	**Obese Restricted body fat**	**Obese No restriction on body fat**
	**n = 197**	**n = 154**	**n = 148**	**n = 180**
	**n (rate/100)**	**n (rate/100)**	**n (rate/100)**	**n (rate/100)**
New injuries	22 (11)	19 (12)	44 (30)	41 (23)
Rate ratio	1 (Reference)	1.1 (0.6, 2.1)	2.7 (1.6, 4.7)	2.0 (1.2, 3.6)
*p*-value	−	0.75	<0.01	<0.01
Cause of injury				
Injuries incurred during physical training	14 (7)	9 (6)	32 (22)	16 (9)
Rate ratio	1 (Reference)	0.8 (0.3, 2.0)	3.0 (1.6, 6.2)	1.3 (0.5, 2.8)
*p*-value	−	0.66	<0.01	0.55
Overuse/exertion	9 (5)	10 (6)	28 (19)	15 (8)
Rate ratio	1 (Reference)	1.4 (0.5, 4.0)	4.1 (1.9, 10.0)	1.8 (0.8, 4.7)
*p*-value	−	0.45	<0.01	0.16
Stumble/fall/trip	9 (5)	7 (5)	10 (7)	17 (9)
Rate ratio	1 (Reference)	1.0 (0.3, 3.0)	1.5 (0.5, 4.1)	2.1 (0.9, 5.3)
*p*-value	−	1.0	0.40	0.08
Severity of injury				
Mild	10 (5)	7 (5)	20 (14)	27 (15)
Rate ratio	1 (Reference)	0.9 (0.3, 2.6)	2.7 (1.2, 6.4)	3.0 (1.4, 6.8)
*p*-value	−	0.84	0.01	0.02
Incapacity <5 days	3 (2)	8 (5)	15 (10)	7 (4)
Rate ratio	1 (Reference)	3.4 (0.8, 20.0)	6.7 (1.9, 35.9)	2.6 (0.6, 15.3)
*p*-value	−	0.06	<0.01	0.18
Consequence of injury				
Absence from physical training/sport	21 (11)	8 (5)	29 (20)	24 (13)
Rate ratio	1 (Reference)	0.5 (0.2, 1.1)	1.8 (1.0, 3.4)	1.3 (0.7, 2.4)
*p*-value	−	0.08	0.03	0.46
Absence from work	5 (3)	7 (5)	11 (7)	16 (9)
Rate ratio	1 (Reference)	1.8 (0.5, 7.2)	2.9 (0.9, 10.8)	3.5 (1.2, 12.2)
*p*-value	−	0.33	0.04	0.01

N.B. Other categories of injuries included recurring injuries, chronic injuries, injuries resulting from strikes/collisions, moderate injuries, severe injuries, incapacity >5 days and no treatment required. For the purpose of brevity, these data are not shown because the prevalence of these types of injuries was comparatively low, and there were no significant differences between the four cohorts.

### Absenteeism and productivity

Data for absenteeism were also adjusted for age (15–19, 20–24, 25–29, 30+ years), sex, service (i.e., army, navy, air force) and rank (i.e., officer, recruit). The number of restricted work days varied, but in general, was not significantly different between the four cohorts (Table 4). The number of full days off work was relatively low in all of the cohorts, so no statistical comparisons were performed. Productivity losses from full days off work were higher in the obese cohorts compared with the normal cohort (Table 5). Compared with the normal cohort, productivity losses from restricted work days were significantly higher (22%) in the obese cohort with no restriction on body fat (p = 0.04), and tended to be higher in the obese cohort with restricted body fat (p = 0.08) (Table 5).

**Table 4:** Absenteeism

	**Normal**	**Overweight**	**Obese Restricted body fat**	**Obese No restriction on body fat**
	**n = 197**	**n = 154**	**n = 148**	**n = 180**
Restricted work days				
Total	3,157	1,562	3,766	4,064
Mean (± SD)	16 ± 60	10 ± 33	25 ± 70	23 ± 58
Ratio of means	1 (Reference)	0.6 (0.3, 1.2)	1.6 (0.8, 3.0)	1.4 (0.8, 2.6)
*p*-value	—	0.15	0.15	0.26
Full days off work				
Total	31	49	83	158
Mean (± SD)	0 ± 1	0 ± 1	1 ± 3	1 ± 5

N.B. Data are adjusted for age, sex, service and rank.

**Table 5:** Costs of healthcare usage and productivity loss from full days off work and restricted work days

	**Cohort**			
	**Normal**	**Overweight**	**Obese Restricted body fat**	**Obese No restriction on body fat**
	**n = 197**	**n = 154**	**n = 148**	**n = 180**
Hospital visits				
Total	$853,847	$856,628	$867,753	$1,239,051
Mean (±SD)	$4,334 (11,783)	$5,563 (13,227)	$5,863 (12,299)	$6,884 (12,717)
Median	$0	$1,391	$1,391*	$1,391*
(Range)	($0 − 11,1250)	($0 − 11,6812)	($0 − 97,344)	($0 − 80,656)
Medical specialist consultations				
Total	$121,861	$125,456	$148,234	$187,300
Mean (±SD)	$619 (781)	$815 (912)	$1,002 (1,049)	$1,041 (1,039)
Median	$355	$440*	$673*	$682*
(Range)	($0 − 4988)	($0 − 4,666)	($0− 6,029)	($0 − 5,521)
Medical scans				
Total	$17,925	$24,375	$28,380	$35,877
Mean (±SD)	$91 (208)	$158 (281)	$192 (303)	$199 (346)
Median	$0	$0*	$42*	$40*
(Range)	($0 − 1,238)	($0 − 1,265)	($0 − 1,617)	($0 − 1,620)
Productivity loss from full days off work (per calendar day)				
Total	$3,015	$5,445	$10,066	$16,994
Mean (±SD)	$15 (91)	$35 (157)	$68 (371)	$94 (496)
Median (Range)	0 ($0 − 881)	0 ($0 − 1,370)	0 ($0 − 4,111)*	0 ($0 − 5,022)*
Productivity loss from full days off work (per work day)				
Total	$4,233	$7,644	$14,131	$23,858
Mean (±SD)	$21 (128)	$50 (220)	$95 (521)	$133 (696)
Median (Range)	0 ($0 − 1,237)	0 ($0 − 1,924)	0 ($0 − 5,771)*	0 ($0 − 7,051)*
Productivity loss from restricted work days (50% limited activity)				
Total	$164,945	$87,997	$175,430	$213,361
Mean (±SD)	$837 (3,135)	$571 (1,852)	$1,359 (3,664)	$1,185 (3,028)
Median (Range)	0 ($0 − 25,806)	0 ($0 − 17,097)	0 ($0 − 22,707)	0 ($0 − 27,943)*

* *p* < 0.05 vs BMI 18.5 − 24.9.

### Cost of healthcare usage

The cost of hospital visits, consultations with medical specialists and medical scans were significantly higher (p < 0.01) in both obese cohorts compared with the normal cohort (Table 5). In contrast, the costs of pathology investigations (p = 0.17) and pharmaceutical prescriptions (p = 0.82) were similar between all four cohorts (data not shown).

### Administrative outcomes

The proportion of individuals who were reclassified to a lower Medical Employment Classification was 9% in the normal cohort, 6% in the overweight cohort, 11% in the obese cohort with restricted body fat and 8% in the obese cohort no restriction on body fat (p = 0.18). The proportion of individuals who were discharged from service (for any reason) was 4% in the normal cohort, 4% in the overweight cohort, 5% in the obese cohort and restricted body fat, and 6% in the cohort with no restriction on body fat (p = 0.69). The probability of discharge was not significantly different between the two obese cohorts (p = 0.90).

## Discussion

This is the first investigation into the health, administrative and economic consequences of high BMI specifically among new recruits in their first year of training in the ADF. The findings of this report are important for several reasons. Military service is unusual compared with many other professions, because it requires a greater level of physical health. High BMI in the military can potentially disrupt military readiness, workforce maintenance and productivity—all of which are integral to effective service provision. The long-term costs of health maintenance and medical care associated with high BMI may place an economic burden on military funding, because the ADF pays for the healthcare costs of its employees. However, if entry into military training is restricted because of high BMI, then this may limit the size and capacity of future military operations.

### Illness

Obesity is a significant risk factor for a variety of chronic diseases [1]. However, over the relatively short duration of active service in the ADF (~7 years), it is unlikely that ADF personnel will develop chronic disease as a result of obesity. They are more likely to experience episodes of acute illness over this time frame. The present data indicate that the prevalence of illness was higher in the obese cohorts compared with the normal cohort. In the general population, obese individuals present with more illnesses [2], and visit general practitioners more frequently for consultations on common illnesses [6] compared with individuals with lower body mass. The reasons why obese individuals are more susceptible to illness are unclear. Metabolic abnormalities associated with obesity may contribute to chronic, low-grade inflammation, which may in turn reduce resistance to infection [24]. Resistance to infection is important among military personnel, who often deploy to foreign regions, where they are exposed to contaminated food and water or environmental toxins. The present data suggest that excess body fat among obese individuals does not increase the risk of illness.

### Injury

Physical injury, whether acute or chronic, is a significant factor influencing training and deployability of military personnel. High BMI is an important risk factor for injury in civilian workplaces [3,4]. Consistent with previous research, the present data indicate that the prevalence of injury was higher among the obese cohorts compared with the normal cohort. [Ross et al. 25] identified that recruits to the Royal Australian Air Force with a high BMI were at greater risk of injury during training compared with those recruits with normal BMI. Other data supporting body fat and/or BMI as a risk factor for injury comes mainly from the U.S. defence force. Knapik et al. have reported that high BMI increases the risk of injury in male, but not female military personnel [26]. In their study of U.S. defence force recruits undergoing basic military training, Cowan et al. observed crude injury rates of 5.2/1000 person-days in ‘over body fat’ individuals compared with 3.6/1000 in ‘weight qualified’ individuals [7]. The present data suggest that among obese individuals excess body fat (i.e., >24% for males and >28% for females) does not increase the prevalence of injury.

In the present study, both obese cohorts suffered more new injuries and mild injuries compared with the normal cohort. The obese cohort with restricted body fat also suffered more injuries resulting from physical training, overuse injuries and injuries resulting in incapacity for <5 days compared with the normal cohort. The greater prevalence of overuse injuries in the obese cohort with restricted body fat compared with the normal cohort is consistent with other reports [27]. Cowan et al. observed that overuse injuries were 47% more likely to occur in U.S. defence force recruits classified as ‘over body fat’ compared with those recruits classified as ‘weight qualified’ [7]. They also noted that the prevalence of sprain/strain, ankle/foot, back and lower leg injuries was greater in recruits classified ‘over body fat’. The most likely reason for these observations is that increased body mass increases strain on soft tissues such as tendons, cartilage and fascia [28-30]. Greater body mass itself may also increase ground reaction forces, which in turn can raise the risk of overuse injuries [31].

Other research on foreign defence force personnel has identified poor physical fitness as a strong predictor of injury among obese individuals [8,26,32]. Low muscular endurance and poor flexibility may contribute directly to injury, whereas low cardiorespiratory fitness may contribute indirectly as a result of greater fatigue and uneconomical performance of tasks [13,33]. High BMI alters body geometry and postural stability [34,35]. In turn, these alterations may reduce movement efficiency and increase the risk of injury [36,37]. In obese army personnel, physical training and lifestyle counselling assists in reducing body mass and fat mass, and improving cardiorespiratory fitness [38,39]. Improvements in physical health and fitness through training help to reduce the risk of injury during basic military training [8,9].

### Absenteeism and productivity

More frequent illness and/or injuries among obese individuals increase healthcare costs and absenteeism. In the civilian population, obesity is associated with greater healthcare costs, more frequent medical consultations and more days away from work [6,33]. Cowan et al. reported that the rate of healthcare utilisation was 10.6/1000 person-days in U.S. defence recruits classified as ‘over body fat’ compared with 3.6/1000 in recruits classified as ‘weight qualified’. This difference translated to a 49% higher rate of utilisation among ‘over body fat’ individuals [7]. In the present study, the obese cohort with no restriction on body fat required more days in hospital days compared with the normal cohort. The prevalence of injuries requiring absence from work and the costs of medical consultations and medical scans were also higher in both obese cohorts compared with the normal cohort. In addition to these financial costs, injury and illness can also result in lost proficiency and work time [40]. In the present study restricted work days were generally similar between all four cohorts. This study did not capture the psychosocial effects of injury and illness on an individual level. Nevertheless, this issue is important to consider, because the psychosocial stress of losing physical ability and an occupational role through injury and illness may influence individual workplace productivity in the long term [40].

Other research has also examined the health and productivity costs of high BMI in foreign defence forces. In the U.S. Navy, over a period of 5 years, the overall annual expense for in-patient care for the 10 most common obesity-related disorders was estimated at more than $5.8 M USD [11]. In the U.S. Air Force, annual medical care expenses among obese personnel were $19.3 M USD, and there was a total of 28,351 lost workdays, which amounted to $3.5 M USD in expenditure [14]. Among Finnish military personnel, increasing BMI correlated significantly with number of sick days [10]. The mean (95% confidence intervals) work disability costs in the group with BMI 26.5 was €710 (€626 − 807) compared with €462 (€445 − 525) in the group with BMI 25.8 [10]. Increased absenteeism among obese individuals may be related to low physical activity, particularly among women [41]. In turn, low physical activity may increase the frequency of sick days by increasing the risk of musculoskeletal injuries [42,43] and upper respiratory illnesses [44].

### Administrative outcomes

Severe acute injuries, or chronic illness and injuries among military personnel with high BMI potentially increase the risk of training restrictions, reclassification and/or discharge from military service [45]. These administrative outcomes resulting from high BMI will likely influence workforce maintenance and the economic costs of recruitment and training. In the present study, despite the higher prevalence of injuries and illnesses in the obese cohorts, the proportion of individuals in these two cohorts who were reclassified to lower Medical Employment Classification and/or discharged was similar to the normal cohort. Other research into whether high BMI increases the likelihood of discharge from military service has produced equivocal findings. Premature discharge rates were highest among obese Swedish military personnel, and low levels of physical activity with a prior history of injuries to the knee and lower back [46]. Taanila et al. [32] observed that discharge on medical grounds was strongly associated with low physical fitness among Finnish military personnel. Packnett et al. reported a clear ‘U-shaped’ relationship between BMI and the risk of all-cause and medical discharge in the U.S. Army. Specifically, discharge was significantly more likely in soldiers with BMI <17 and BMI >30 [12]. Poor physical health may increase the risk of medical discharge not only through injury, but also through greater feelings of depression and anxiety [46].

Several issues arising from the present study warrant further discussion. First, this study highlights some of the short-term consequences of high BMI in the military. However, the health consequences of high BMI are more likely to become evident in the longer term when military personnel are veterans. Second, individuals in the overweight and obese cohorts were older on average than individuals in the normal cohort. The differences between these cohorts may therefore result not only from greater body mass, but other factors, possibly including advancing age. Third, health outcomes and healthcare usage were generally similar between the obese cohorts. Paradoxically, some aspects of physical injury were actually lower in the obese cohort with no restriction on body fat compared with the cohort with restricted body fat. This finding suggests that increased body mass, and not body fat *perse*, influences health outcomes and healthcare usage in obese military personnel. Last, the validity of BMI assessment on its own as a criterion for entry into military service is questionable. Several studies in military [17,18] and athletic populations [19] indicate that BMI can overestimate or underestimate percent body fat. Other methods for assessing body composition (e.g., bioelectrical impedance, dual energy x-ray absorptiometry) can provide more accurate estimates for percent body fat [17,18]. However, it is often impractical to use these methods when screening applications for entry into military training—particularly if applicants reside in various locations. Therefore, although defence forces around the world are likely to retain BMI as a screening tool, BMI should be interpreted with caution. A further consideration for screening applications to enter military training is that body fat itself is also not necessarily an appropriate measure of health status [47].

## Conclusions

In summary, the present study demonstrates that for young men and women entering military training, obese individuals experienced more illnesses and injuries, and incurred greater medical costs (e.g., specialist consultations, scans) compared with those individuals with normal BMI. Among obese individuals, the prevalence of injury and illness, healthcare usage and productivity were not significantly greater in the cohort with no restriction on body fat. Obese individuals did not require more time off work and were no more likely to be classified as unfit for service or discharged from service. These findings therefore suggest that high BMI may raise the costs of healthcare, but does not disrupt workforce maintenance.

## Abbreviations

ADF: Australian Defence Force; BMI: Body mass index.

## Competing interests

The author(s) declare that they have no competing interests.

## Authors’ contributions

JP carried out data entry and analysis and prepared the manuscript. SG collected figures for the economic modelling and helped to prepare the manuscript. MW performed the data analysis and helped to prepare the manuscript. RM designed the study, obtained funding, and assisted with data collection. TG assisted with data collection and helped to prepare the manuscript. GW designed the study and helped to prepare the manuscript. PN designed the study, obtained funding, assisted with data collection, advised on the economic modelling and helped to prepare the manuscript. PW helped to prepare the manuscript. All authors have read and approved the final manuscript.

## Pre-publication history

The pre-publication history for this paper can be accessed here:

http://www.biomedcentral.com/1471-2458/12/451/prepub
